# Serum Exosome MicroRNA as a Minimally-Invasive Early Biomarker of AML

**DOI:** 10.1038/srep11295

**Published:** 2015-06-12

**Authors:** Noah I. Hornick, Jianya Huan, Ben Doron, Natalya A. Goloviznina, Jodi Lapidus, Bill H. Chang, Peter Kurre

**Affiliations:** 1 Department of Pediatrics, Oregon Health & Science University, Portland, OR; 2 Department of Medicine, Oregon Health & Science University, Portland, OR; 3 Department of Public Health, Oregon Health & Science University, Portland, OR; 4 Papé Family Pediatric Research Institute, Oregon Health & Science University, Portland, OR; 5 Knight Cancer Institute, Oregon Health & Science University, Portland, OR.

## Abstract

Relapse remains the major cause of mortality for patients with Acute Myeloid Leukemia (AML). Improved tracking of minimal residual disease (MRD) holds the promise of timely treatment adjustments to preempt relapse. Current surveillance techniques detect circulating blasts that coincide with advanced disease and poorly reflect MRD during early relapse. Here, we investigate exosomes as a minimally invasive platform for a microRNA (miRNA) biomarker. We identify a set of miRNA enriched in AML exosomes and track levels of circulating exosome miRNA that distinguish leukemic xenografts from both non-engrafted and human CD34+ controls. We develop biostatistical models that reveal circulating exosomal miRNA at low marrow tumor burden and before circulating blasts can be detected. Remarkably, both leukemic blasts and marrow stroma contribute to serum exosome miRNA. We propose development of serum exosome miRNA as a platform for a novel, sensitive compartment biomarker for prospective tracking and early detection of AML recurrence.

Acute Myeloid Leukemia (AML)^1^ causes more than 10,000 deaths annually in the United States, with a 5-year overall survival rate of approximately 25%^1^. Induction treatment of AML achieves disease remission in up to 80% of patients, yet a majority among adults and children experience relapse. This, along with the aggressive kinetics of relapsing AML (2.5 log/month in one recent study^2^), underscores the need for improved early detection of residual disease after induction chemotherapy. Indeed, end of induction MRD is the most powerful marker of poor outcome for childhood AML^3^ and alters clinical decision making^2^,^4^. However, current methodologies do not perform well at later time points, in part because conventional detection of relapse is uniformly based on identifying leukemia at the cellular level^5^. Peripheral blood assays require the presence of leukemic blasts in the circulation, which generally connotes advanced disease burden, while bone marrow aspirates are invasive and rely on a sample from a single physical location. Ineffective surveillance strategies are illustrated by a recent retrospective study on pediatric AML that found that forty-one routine bone marrow aspirates were performed for every case of relapse detected^6^. Additionally, both flow cytometry and PCR require the presence of a known leukemia-specific marker. Unlike *BCR-ABL1* in chronic myeloid leukemia, AML lacks a molecular marker whose detection specifically identifies leukemia. Combined, these features have to date precluded the development of a clinically useful and timely prospective surveillance strategy.

Cytokines^7^ and circulating cell-free nucleic acids^8^,^9^ have been explored for MRD tracking, and more recently, microRNA (miRNA) have attracted attention as a potential source of novel AML biomarkers^10^,^11^. MicroRNA expression profiles have been associated with AML subtypes^12^, mutations^13^, and overall survival^14^. Exosomes – small, membrane-enclosed extracellular vesicles – are directly secreted by AML blasts^15^, carrying a select panel of cellular RNA and protein^16^,^17^,^18^. Exosomes equilibrate between tissue compartments and can be isolated from many body fluids^19^,^20^, including both plasma^21^ and serum^22^; and while their potential as biomarkers is beginning to be explored^22^,^23^,^24^, exosome miRNA have not yet been investigated as a marker of AML disease burden. Based on our recent findings that AML blasts secrete exosomes that contain selected miRNA^15^, we hypothesized that leukemia patient serum contains exosomes bearing miRNA that uniquely identify AML, providing potential advantages in both sensitivity and specificity over conventional biomarkers based on direct measures of plasma-associated miRNAs^25^.

## Results

### NSG xenograft of Molm-14 produces a model of AML disease with characteristic exosome secretion

**Figure 1:**
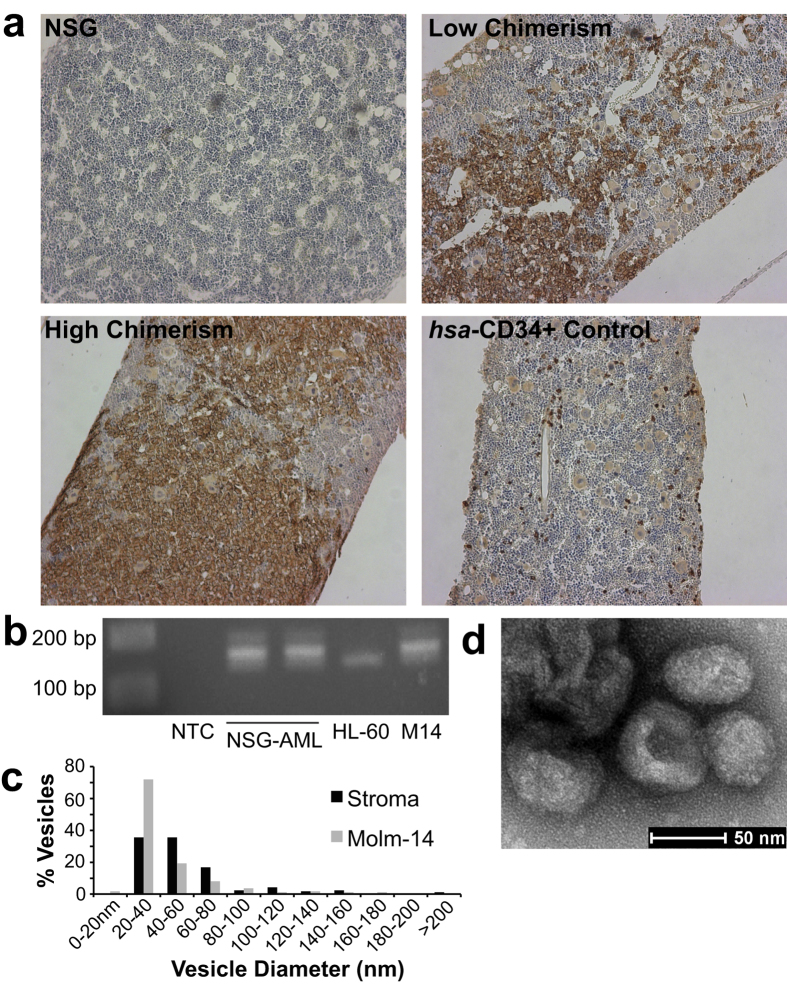
Molm-14 Engraft in NSG Mice and Contribute to Compartment Exosome Secretion (**a**) Immunohistochemistry of Molm-14-engrafted NSG femurs. Femurs were collected from Molm-14-engrafted mice at 3 weeks post-engraftment, sectioned, and stained for human CD45. (**b**) PCR identification of NSG-engrafted AML. Molm-14 isolated from engrafted NSG mice retained their characteristic FLT3-ITD mutation, detected by 40 cycles of PCR (cropped; full gel in Figure S2). NTC: null-template control; NSG-AML: isolated Molm-14; HL-60: cell line exhibiting WT FLT3; M14: Molm-14 stock culture. (**c**,**d**) Transmission electron microscopy of exosomes. Exosomes were isolated from the culture media of Molm-14 and NSG stroma, and examined by transmission electron microscopy. Sizing of more than 100 vesicles per sample is presented in (**c**); representative stromal exosome image is presented in (**d**).

As a model for high-risk AML with rapid growth kinetics, we developed a xenograft model using the Molm-14 cell line in NOD/SCID/IL-2rγ^null^ (NSG) mice^26^,^27^,^28^. Upon tail vein injection of as few as 1 × 10^5^ Molm-14 cells, we found predictable establishment of disease in NSG mice. We avoided irradiation conditioning in order to maintain vascular integrity and the native microenvironment. Engrafted animals were sacrificed at serial time points and human CD45 was readily detected in sectioned femurs by immunohistochemistry (Fig. 1a). In a series of experiments comprising leukemias in more than 30 animals, we found that disease was rapidly progressive, leading to death or morbidity requiring sacrifice within a 4-week time frame. In order to establish the source of exosomes within the leukemia niche, we isolated leukemic cells from mice engrafted with Molm-14 AML (bone marrow chimerism between 69–77% by human CD45 positivity). Using PCR for FLT3-ITD verified that these animals contained leukemic cells similar to Molm-14 stock (Fig. 1b, Fig. S2). As the bone marrow microenvironment promotes leukemic growth, proliferation, and anti-apoptotic signaling, and has been shown to confer chemoresistance in NSG models^29^,^30^,^31^, we additionally isolated stroma by adhesion of cells from whole bone marrow to culture flasks, and then evaluated them in culture after 4–6 passages. Expansion cultures of both murine stromal cells and NSG-engrafted Molm-14 produced exosomes with typical size (Fig. 1c) and morphology (Fig. 1d). These results indicate that both leukemic cells and marrow stroma can contribute to serum exosomes, providing a potential compartment-level indicator of disease.

### AML cell- and AML-conditioned stroma-secreted exosomes carry a select miRNA subset

**Figure 2:**
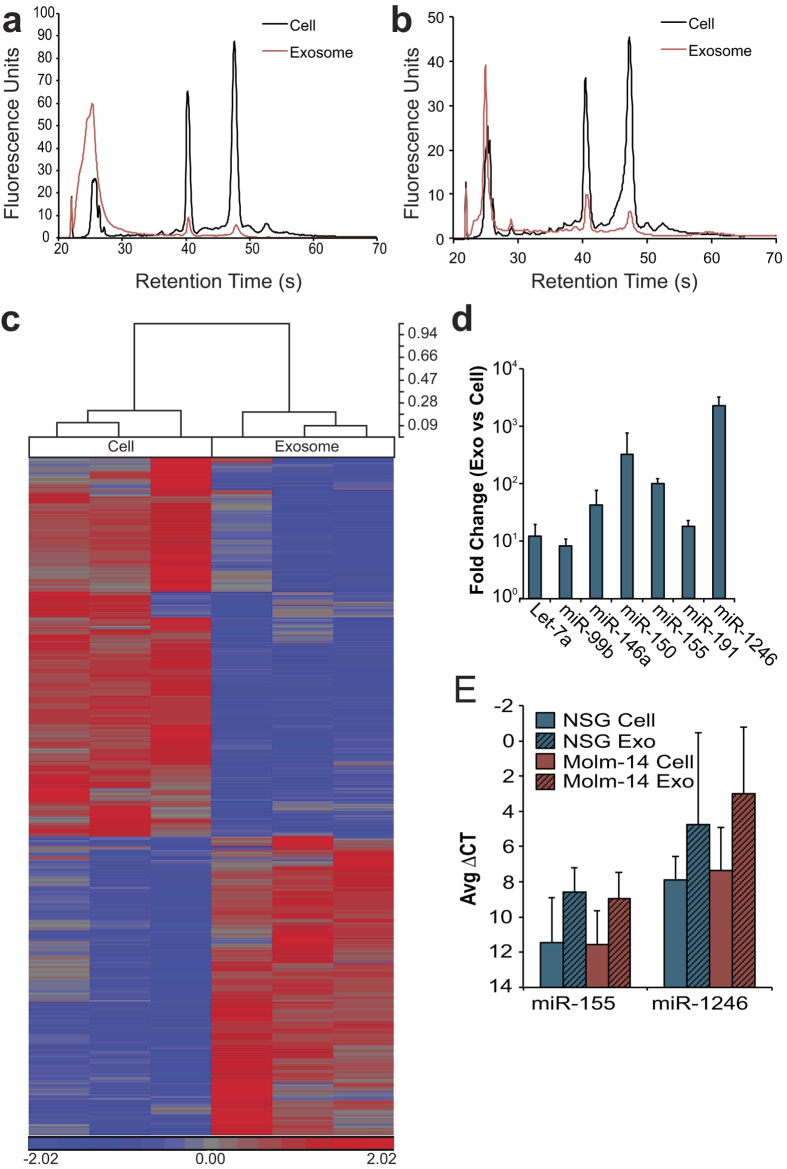
Molm-14 and Stromal Exosomes Concentrate Selected MicroRNA (**a**,**b**) RNA size profiles of exosomes. RNA was collected from Molm-14 (**a**) and NSG Stromal (**b**) cells and their exosomes after 72 hours in culture, and miRNA was evaluated using a bioanalyzer. (**c**) Microarray comparison of cell, exosome miRNA. Molm-14 cell and exosome microRNA was evaluated using an Affymetrix microRNA microarray. All targets with more than 2-fold mean difference between producing cell and exosome are represented, RMA-corrected and standardized to a mean of 0 and a SD of 1. Dendrogram values are 1 - Pearson’s R. (**d**,**e**) qRT-PCR for miRNA in cells *versus* exosomes. Selected targets from NSG stromal (**d**) and Molm-14 (**e**) cells and exosomes were validated using Taqman qRT-PCR, normalized to U6 snRNA. Fold change was calculated by 2^-ΔΔCt.

We previously showed that AML-secreted exosomes, including those from Molm-14, contain a concentrated miRNA population that is proportionally different from the cellular miRNA^15^. In extension of those observations, we found an enrichment of small RNA within the total RNA of exosomes derived from *ex vivo* Molm-14 after engraftment in NSG mice (Fig. 2a) as well as exosomes derived from NSG stroma, (Fig. 2b) when compared to the RNA from cell of origin. As miRNA are highly conserved^32^, and thus unlikely to be unique to a particular disease process (or species), we pursued a wider characterization of the respective cellular / exosomal miRNA levels in order to determine whether an predictive panel could be identified^8^. We evaluated Molm-14 exosomal *versus* cellular miRNA using the Affymetrix GeneChip miRNA 3.0 microarray (GEO Accession GSE55025), containing almost 20,000 unique probes based on miRBase v17. This revealed stark differences between the miRNA content of exosomes and the cells that produce them (Fig. 2c). Of the mature miRNAs detected in both cell and exosome, 161 of 1733 were at least two-fold increased in exosomes, while 108 were at least 2-fold decreased. We used quantitative reverse transcriptase-PCR to validate our microarray findings for select targets noted in the recent literature to be relevant to AML biology^33^,^34^. After normalizing to U6, we found substantially enriched concentrations of let-7a, miR-99b, -146a, -155, -191, and -1246, concentrations of up to 1000-fold above cellular levels (Fig. 2d). We also evaluated several of these miRNA in exosomes isolated from *ex vivo* stromal cell cultures (Fig. 2e). Although these miRNA tended to be abundant in exosomes compared to the producing stromal cells, there was no significant difference between cell and exosome, nor between stroma from Molm-14 engrafted animals and controls. The array studies provided us with a candidate panel of miRNA targets, derived from both AML and its surrounding stroma, to pursue in our investigations into total serum exosome miRNA profiles.

### Conventional surveillance strategies provide a limited window into leukemic burden

**Figure 3:**
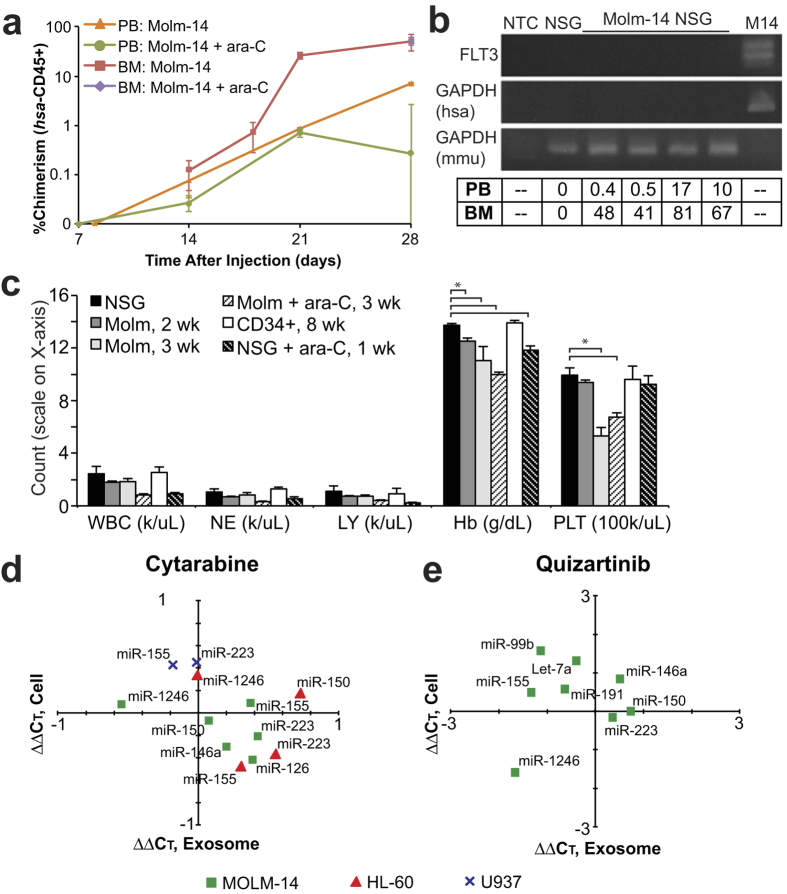
Conventional Biomarkers Provide Limited Information; Chemotherapy is a Confounder (**a**) Molm-14-engrafted NSG chimerism by flow. Flow cytometry for human CD45 was performed on the peripheral blood and bone marrow of Molm-14-engrafted NSG mice, both without and with cytarabine treatment (300 mg/kg, Q3Dx3 starting 14d post-engraftment). (**b**) mRNA markers of AML in serum exosomes. Exosomes isolated from the peripheral blood of Molm-14-engrafted NSG mice were tested for human FLT3 and GAPDH and murine GAPDH using 45 cycles of PCR (cropped; full gels in Figure S3). NTC: null-template control. M14: Molm-14 stock culture. PB: Peripheral blood chimerism; BM: Bone marrow chimerism by flow cytometry for human CD45. (**c**) CBC of Molm-engrafted NSG. Peripheral blood CBC of mice, comparing baseline NSG (n = 31), NSG engrafted with Molm-14 at 2 (n = 16) and 3 (n = 5) weeks post-engraftment, NSG engrafted with Molm-14 and treated with cytarabine (n = 8) at 3 weeks, control NSG given cytarabine (n = 4), and NSG given nonmalignant CD34+ human cells (n = 4). WBC, white blood cells; LY, lymphocytes; NE, neutrophils; Hb, hemoglobin; PLT, platelets (×1000/uL). *, *p* < 0.05 by Student’s *t*. Error bars represent SEM. (**d**,**e**) *In vitro* chemotherapy and exosomal miRNA. Molm-14, HL-60, and U937 cells were exposed to cytarabine (25 ng/mL, **d**) overnight, and Molm-14 were exposed to quizartinib (0.1 nM, **e**) overnight. Cellular (on the x-axis) and exosomal (on the y-axis) levels of miRNA were measured by qRT-PCR, and are presented as ddCT (a -1 ddCT represents a 2-fold increase in miRNA).

For reference to existing leukemia monitoring strategies, we evaluated flow cytometry and complete blood count (CBC) in our model. Flow cytometry on PBMCs proved to be an unreliable means of detection; disease was first reliably detectable by this method (using the threshold of 0.3% human CD45 positivity^35^) within two days of the earliest animal death, reflecting human disease and speaking to the vascular integrity of the BM sinusoids and the physiology of the model. Across multiple experiments, human CD45 chimerism in the marrow developed approximately seven days earlier than in the periphery (Fig. 3a). To track disease burden through treatment, we used cytarabine (ara-C), a nucleoside analog and backbone of AML chemotherapy^36^. Treatment with cytarabine effectively eliminated Molm-14 cells from the blood. Leukemic chimerism within the marrow, however, was unaffected (Fig. 3a), underscoring the problems inherent in the reliance on peripheral leukemia cells as an indicator of disease burden. When we followed complete blood count measures of hemoglobin and platelet counts we found significant differences (*p* < 0.05) in Molm-14 engrafted versus control animals as early as two weeks after engraftment (Fig. 3c), at which time peripheral blood chimerism by flow cytometry was still zero (Fig. 3a). As anticipated, CBC metrics were globally suppressed following treatment of mice with ara-C, illustrating one of several confounders in a clinical post-treatment scenario. Illustrating the more complex situation in patients, however, treatment of unengrafted NSG mice resulted in a significant suppression of hemoglobin measures, effectively mimicking the anemia seen in engrafted animals (Fig. 3c). As Molm-14 express FLT3-ITD, we investigated whether this established MRD marker transcript was detectable in exosomes isolated from the serum of engrafted mice (Fig. 3b, Fig. S3). Although murine GAPDH was readily found in the serum exosomes of both engrafted – with peripheral blood (PB) and bone marrow (BM) chimerism ranging from 0.4–10% and 41–67% respectively – and control animals, neither Molm-14-specific FLT3-ITD nor human-specific GAPDH was detectable after 45 cycles of PCR, broadly consistent with the comparatively lower abundance of larger RNA species by bioanalyzer. We therefore evaluated the enriched exosomal miRNA population in pursuit of biomarker candidates. As a prerequisite for a robust biomarker, AML exosome miRNA must either be unaltered by exposure of the cells to chemotherapy, or be shifted in a systematic and predictable way; this requirement complicates leukemia surveillance with CBC and flow cytometry. In order to determine whether AML exosomal miRNA was sensitive to drug treatment, we exposed several AML cell lines to chemotherapeutics *in vitro*, and then evaluated the resulting changes in cellular and exosomal miRNA. In a series of experiments in which we exposed multiple AML cell lines to cytarabine, we found that no candidate miRNA exhibited a shift of 2-fold or more after ara-C exposure (Fig. 3d). Similarly, exposure to the targeted kinase inhibitor Quizartinib (which inhibits FLT3 kinase activity^37^) was associated with only minor shifts in exosomal miRNA levels (Fig. 3e). These findings support exosomal miRNA as a biomarker that is representative of disease burden, rather than fluctuating expression or exosome release modified as a result of treatment.

### Exosomal miRNA in the serum echoes AML-engraftment kinetics

**Figure 4:**
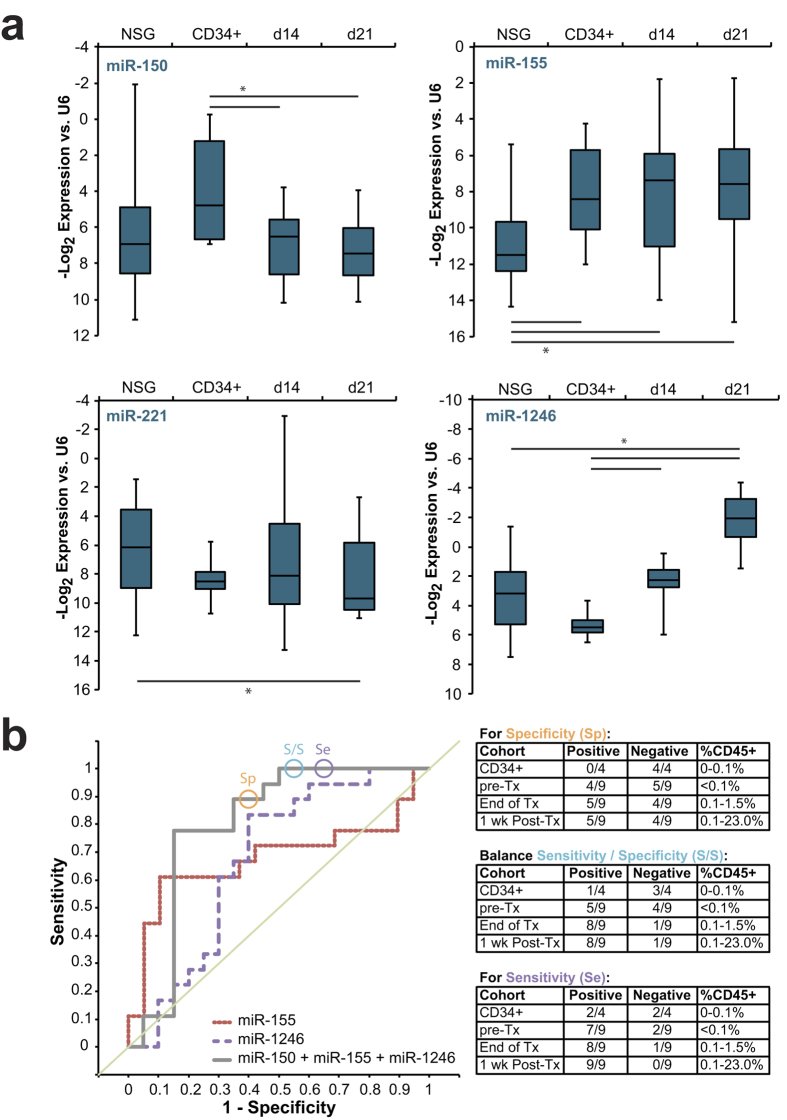
Serum Exosome miRNA Distinguishes Leukemia From Homeostatic Hematopoiesis (**a**) Serum exosome miRNA levels. Exosomes were isolated from peripheral blood of control NSG mice as well as NSG mice engrafted with Molm-14 (after 14 or 21 days; d14 and d21, respectively) or human CD34+ cells. MicroRNA levels were measured by qRT-PCR. *, *p* < 0.05 by Student’s *t*. (**b**) Serum exosome miRNA score performance. Receiver operating characteristic (ROC) curves are presented for single miRNA and for the combination of miRNA-150, -155, and -1246. The circles represent points on the ROC curves whose alphas were chosen as cutoff values emphasizing specificity, sensitivity, or a balance of the two, respectively. These cutoff values and the coefficients generated by the regression were used to evaluate mice engrafted with Molm-14 and treated with cytarabine, alongside control mice engrafted with human CD34+ cells. Exosome miRNA panel performance for each cohort and cutoff is presented alongside peripheral blood chimerism (by flow cytometry for human CD45).

To develop an *in vivo* exosome biomarker platform, we tested quality and quantity of miRNA isolated from small amounts of murine serum. We collected blood from Molm-14 xenograft and control NSG mice and extracted exosomes from the serum. Assessing the amount and quality of RNA yielded in exosome preparations from varying volumes of serum established that exosomal miRNA could be collected from as little as 20 uL of serum (Fig. S1). Based on this finding, we settled on a serum volume of 20–50 μL for exosomal miRNA measurements. In a series of experiments comprising more than sixty mice, we systematically determined serum levels of four candidate miRNA chosen for their relevance to leukemia^25^,^33^,^34^ and incorporation in exosomes (Fig. 1): miR-150, miR-155, miR-221, and miR-1246. Using Molm-14-engrafted animals at 14 days (d14) and 21 days (d21) post-engraftment as our experimental groups, we compared to unengrafted NSG mice as well as to mice engrafted with healthy human CD34+ cells derived from cord blood. In spite of the anticipated substantial inter-animal variability, this panel of miRNA reproducibly distinguished between cohorts (Fig. 4a): miR-155 was elevated in Molm-14- and CD34+-engrafted animals but not NSG controls, miR-150 separated Molm-14 from CD34+ engraftment, miR-221 was altered at late but not early leukemia time points, and miR-1246 increased over time in leukemia-, but not nonmalignant CD34+ cell-engrafted mice. In an effort to test statistical strength, models of receiver operating characteristic (ROC) were generated by logistic regression. These highlight the added discriminatory capacity conferred by combining the top performing markers (Fig. 4b). By evaluating multiple combinations, we developed a model that produces a single exosomal miRNA score (linear combination of miR-150, -155, and -1246) which was best able to separate leukemia-engrafted mice from controls, achieving an area under the curve (AUC) of 0.80, compared to individual marker AUC’s of 0.68 for miR-155 and 0.67 for miR-1246. (Fig. 4b, Table S4). These models were generated using data collected from mice 14 days post-engraftment, in whom peripheral blood leukemic blasts ranged between undetectable to less than 0.1% by flow cytometry for human CD45 positivity. Using the coefficients calculated for the combined miR-150/ -155/ -1246 model generated using day 14 data (−0.2175, −0.3837, and 0.0017, respectively) we scored serum exosome miRNA from a separate validation set of animals engrafted with human cord blood-derived CD34+ cells or with Molm-14, then treated with ara-C at 14 days post-engraftment as described above. In order to determine the potential discriminatory capacity of a set cutoff value, we sorted the scores generated by measuring serum exosomal miRNA in the validation set. We then chose three separate scores (circled in Fig. 4b) statistically emphasizing sensitivity, specificity, or a balance of the two, based on the observed values in this cohort. Optimizing sensitivity or specificity allowed complete elimination of false negatives or false positives, respectively, while balancing the two in choosing a cutoff value resulted in detection of 8/9 leukemias with only 1/4 controls being scored positive (Fig. 4b). These results demonstrate both the reproducibility of alterations in serum exosomal miRNA as a marker of leukemia and the independence of this score from both treatment and the level of circulating blasts.

### Exosomal miRNA as a general AML biomarker

**Figure 5:**
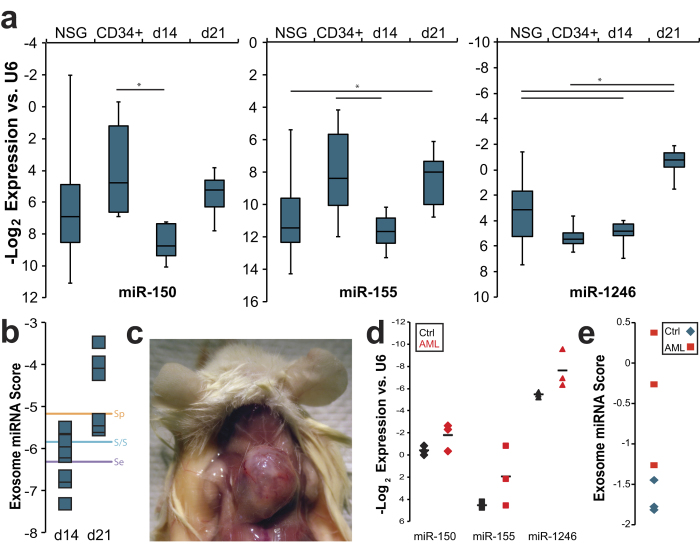
Expansion of the Scope of the Serum Exosome miRNA Biomarker (**a**) Comparing serum exosome miRNA levels in HL-60. Exosomes were isolated from peripheral blood of control NSG mice as well as NSG mice engrafted with HL-60 (after 14 or 21 days; d14 and d21, respectively) or human CD34+ cells. MicroRNA levels were measured by qRT-PCR. *, *p* < 0.05 by Student’s *t*. (**b**) Evaluation of score performance. miRNA panel scores were calculated for HL-60-engrafted mice at 14 and 21 day time. The lines correspond to alphas chosen as described in Fig. 4b. (**c**) Chloromas in HL-60-engrafted NSG mice. (**d**) Patient serum / plasma exosome miRNA. Exosomes were isolated from serum or plasma from AML patients (red) or normal subjects (black), and miRNA levels of miR-150, -155, and -1246 were measured. Means are represented as horizontal bars. (**e**) Evaluation of score in human samples. miRNA panel scores were calculated for the serum miRNA levels depicted in **d**.

In order to determine the broader preclinical potential for exosome miRNA as biomarkers we interrogated an additional model of promyelocytic AML by xenografting HL-60 cells into NSG mice. This corroborated the significant differences between serum exosome levels of miR-150, -155, and -1246 in leukemia engrafted mice and levels in baseline NSG or in human CD34+ cell-engrafted controls (Fig. 5a). As in our Molm-14 xenograft experiments, individual miRNA levels provided variable contributions to the ability to discriminate between cohorts. We therefore applied the scoring algorithm we derived from our Molm-14 xenografts to the miRNA levels obtained from HL-60 xenografted mice (Fig. 5b). When we applied the three cutoffs described in Fig. 4b, we found that the sensitivity / specificity balanced cutoff correctly predicted 100% of HL-60 engrafted animals at 3 weeks post-engraftment; a time point at which human CD45 could not be identified by flow cytometry in 5/7 animals (Table S2). Interestingly, we found that a majority of animals engrafted with HL-60 developed peripheral chloromas (Fig. 5c), in addition to, but primarily in lieu of bone marrow disease, further highlighting the potential for detecting extramedullary disease.

We had the opportunity to investigate the feasibility of our biomarker panel in a small set of patient plasma and serum samples, both previously validated as equivalent sources of circulating miRNA^38^. Evaluating three AML patients and three normal subjects, we found that all three individual exosome miRNA in our panel were detectable in circulating human exosomes, and were markedly different in the AML patients compared to the normal subjects (Fig. 5d). We calculated panel scores from these miRNA levels, which resulted in a clear cutoff between patients and controls (Fig. 5e). This difference tended toward, but did not reach significance (*p* = 0.057 for the overall miRNA panel score), reflecting the small sample size. We consider this result encouraging for further development of exosome miRNA panels to be tested prospectively in AML patient serum.

## Discussion

The substantial morbidity and mortality experienced by AML patients in relapse creates an urgent need for efficient and cost-effective MRD surveillance. Whereas chemotherapy effectively eliminates peripheral AML blasts, most treatment regimens fail to eradicate residual leukemic cells within the bone marrow microenvironment^30^,^31^. In an effort to overcome the limitations of compartmental relapse and reliance on measuring malignant cells directly, we set out to develop exosomal miRNA, as biomarkers of disease, a prospect not limited to AML^22^,^24^,^39^.

Detection of serum exosome miRNA circumvents both the need for an invasive marrow aspiration procedure and the reliance on the presence of leukemic blasts in the periphery. While the endothelium presents a barrier to leukemia cell egress from the marrow^40^, exosomes are able to equilibrate with the bloodstream^41^, and thus are detectable in systemic circulation. Detection of these exosomes peripherally, therefore, could potentially provide evidence of early (including extramedullary) leukemia relapse. Several recent publications indicate a correlation of select circulating miRNA, singly or as a panel of markers, with disease burden in CLL and GVHD^8^,^42^. These tools rely either on direct measures of cellular miRNA, or on total cell-free miRNA in plasma, and have unfortunately not yet produced clinically useful biomarkers^43^. Analysis of circulating exosomes allows evaluation of a population to which AML is known to directly contribute, excluding such populations as HDL-associated and protein-bound miRNA^44^.

Xenotransplantation of human cells into immunodeficient mice, in particular NOD/SCID/IL-2rγ^null^ (NSG) mice, is well established as a model for the systematic *in vivo* study of human AML biology^26^,^27^. Molm-14, a cell line derived from a patient with monocytic leukemia, is positive for the FLT3-ITD mutation^28^, a negative prognostic indicator^45^ and characteristic of an aggressive, rapidly progressive disease. In earlier studies, we had established that Molm-14 and HL60 cells, like those of primary patient-derived AML samples, produced exosomes rich in miRNA^15^. We therefore selected these cell lines to provide consistency for development of this biomarker platform not present using primary AML xenografts with more variable kinetics^46^.

Our recent finding that exosomes transport a preponderance of miRNA, and that the miRNA cargo is not simply a random sampling of cellular content^15^ supports the view of AML-derived exosomes as containing specifically selected miRNA populations. Accordingly, our experiments demonstrate reproducible miRNA perturbations in peripheral blood exosomes, while less abundant AML-specific mRNA transcripts could not be detected in AML exosomes^15^, even with substantial peripheral blood chimerism. In a series of microarray and qRT-PCR experiments we were able to combine multiple measures to derive a panel of miRNA that were not only exported in exosomes by AML, but correlated with the presence of leukemia (Figs 2,4). While miR-1246 and miR-155 individually correlated well with AML in our model (Fig. 4), the analysis of multiple miRNA in combinations allowed us to arrive at the subset most representative of AML disease status. We show that careful analysis of this panel of miRNA, rather than reliance on a single target miRNA level to indicate disease improves both sensitivity and specificity (Fig. 4). When directly compared to the immunophenotypic MRD thresholds of 0.1%, commonly used with AML bone marrow samples, or 0.015% with AML peripheral blood samples, our panel of markers was equally sensitive in Molm-14-engrafted mice and more sensitive in HL-60-engrafted mice, often detecting leukemia in mice with zero chimerism by flow (Figs 4b,5b). These observations are echoed in patient serum exosome miRNA samples that similarly varies between leukemia patients and controls. These results support the position that serum exosomal miRNA can add sensitivity and specificity to the minimally invasive detection of residual or recurrent AML, conferring additional advantage as a cell-free marker unaffected by chemotherapy. Intriguingly, our data indicate that this serum exosome population may reflect contributions from both leukemic blasts and marrow stromal cells modified by the presence of the malignancy. While other multi-component biomarkers have been identified^47^ (including, potentially, circulating cell-free nucleic acid^9^,^25^), to our knowledge this is the first such leukemia biomarker to represent multiple components of the malignant microenvironment.

In aggregate, we demonstrate the feasibility of isolating exosomal miRNA from small volumes of murine serum, allowing for discrimination between AML-bearing and control NSG mice. Through comparison with existing metrics over the course of disease and treatment, we establish exosomal miRNA as a sensitive and robust indicator in our xenograft model. A small set of primary patient samples suggests feasibility and discriminatory potential of this marker as a clinical tool. These experiments provide a platform for the development of clinical AML biomarkers with the potential to provide improved detection of occult disease in a minimally invasive methodology.

## Materials and Methods

### Vertebrate ethics

Primary human CD34^+^ hematopoietic progenitor cells and primary patient and control serum / plasma were collected in accordance with an OHSU IRB-approved protocol in accordance with the ethical guidelines maintained by that committee. All mice were maintained in a pathogen-free barrier facility and experiments were carried out in accordance with an OHSU IACUC-approved protocol in accordance with the ethical guidelines maintained by that committee.

### Cell culture

The AML cell lines U937, HL-60, and Molm-14 were cultured in RPMI media (Life Technologies) with 10% FBS in a Thermo Scientific cell culture incubator at 5% CO_2_. For Molm-14 xenografts, Molm-14 cells were cultured at 1% or 21% O_2_ in equal numbers before engraftment. Cell lines were obtained as previously reported^15^; Molm-14 were authenticated by PCR for characteristic FLT3-ITD prior to engraftment. CD34+ cells were purified from cord blood using MACS cell separation (Miltenyi Biotec) and cultured in serum-free expansion media (StemCell Technologies) supplemented with 100 U/ml penicillin/streptomycin, 40 ng/ml Flt-3, 25 ng/ml SCF, and 50 ng/ml TPO (Miltenyi Biotec). Primary murine stromal cells were BM stromal cells were obtained by culturing whole BM cells isolated from the experimental animals in IMDM (Life Technologies) with 20% Vesicle-free FBS^15^ for 4 days, then washing 2x with versene.

### Mice and xenografts

NOD/SCID/IL-2rγ^null^ mice (NSG) were purchased from The Jackson Laboratory. Animals 6–8 weeks old were used in the experiments. 1 × 10^5^ Molm-14 cells/human cord blood-derived CD34^+^ cells or 5 × 10^6^ HL-60 cells per animal were engrafted into non-irradiated animals by *i.v*. tail-vein injection. Retroorbital blood draws were conducted as necessary to obtain serum exosomes or peripheral blood for human CD45 chimerism analysis by flow cytometry. Animals were sacrificed at indicated time points, when peripheral blood, spleen, and bone marrow were collected from each animal. For cytarabine-treated animals, ara-C was administered by intraperitoneal injection at 300 mg/kg every third day for three total injections^48^ starting 14d post-engraftment.

### Exosome preparation and RNA extraction

Exosomes from *in vitro* cultures were isolated by differential centrifugation as described previously^15^. Briefly, AML cells were cultured for 48 hrs, the culture media was spun at 300 × *g* for 10 min to remove cells, then the supernatant was spun at 2,000 × *g* for 20 min and 10,000 × *g* for 20 min to remove cell debris. The supernatant was centrifuged at 100,000 × *g* for 2 hrs to pellet exosomes. Exosomes from NSG serum were extracted using ExoQuick (System Biosciences). Although polymer-based exosome extraction technologies (such as ExoQuick) may co-precipitate other proteins and vesicles, we selected ExoQuick as a translatable means of obtaining enriched exosome-derived RNA from small-volume biological samples; an approach validated by other researchers^49^. RNA was extracted from exosomes or cells using the miRNeasy kit (Qiagen) according to manufacturer’s instructions.

### RNA analysis and qRT-PCR

RNA was extracted from exosomes and cells using miRNeasy or RNeasy kits and quantified using a Nanodrop 2000c (Thermo). RNA integrity was measured using the Agilent Bioanalyzer ‘Pico Chip’ (Agilent). For RT-PCR, RNAs were converted into cDNA using the SuperScript III First Strand Synthesis Kit (Invitrogen) with oligo-dT priming, followed by PCR. A SYBR Green PCR kit (Applied Biosystems) was used for quantitative PCR.

### Immunohistochemistry (IHC) staining

Femur IHC staining for human CD45 was performed by the Histology Core Facility at the Oregon Health and Science University. In brief, a femur was removed from each experimental mouse and fixed in 10% formalin for 24 hrs at 4 °C, then transferred to cold 70% ethanol for the storage until sectioning. The fixed femur was embedded in paraffin and sectioned at 5-μm thickness. The femur slides were rehydrated and high-pressure treated. The treated slides were blocked and then stained with mouse anti-human CD45 antibody (Clone HI30, BioLegend) at 1:150 dilution and then biotinylated anti-mouse IgG. Mouse IgG1 isotype was used as the control. The images of antibody-stained slide were taken with a Leica ICC50 microscope equipped with HD camera using 20X lens. The image processing was performed using Adobe Photoshop (Adobe).

### Flow cytometry analysis

Murine peripheral blood or bone marrow cells were labeled with antibodies to murine CD45 (Clone 30-F11) and human CD45 (Clone HI30, BioLegend) and analyzed by FACSCalibur (BD Biosciences). All data was analyzed using FlowJo software (Tree Star).

### Transmission Electron Microscopy

10 μl of Exosome preparations were deposited onto glow discharged carbon formvar 400 Mesh copper grids (Ted Pella 01822-F) for 3 min, rinsed 15 secs in water, wicked on Whatman filter paper 1, stained for 45 secs in filtered 1.33% (w/v) uranyl acetate, wicked and air dried. Samples were imaged at 120 kV on a FEI Tecnai™ Spirit TEM system. Images were acquired as 2048 × 2048 pixel, 16-bit gray scale files using the FEI’s TEM Imaging & Analysis (TIA) interface on an Eagle™ 2K CCD multiscan camera.

### Microarrays

Microarray assays were performed in the OHSU Gene Profiling Shared Resource. For each sample, a 130 ng of total RNA was labeled using the Flash-Tag Biotin HSR miRNA Labeling Kit (Affymetrix) by polyadenylation and ligation with biotinylated 3’DNA dendrimers. Labeled RNA was mixed with hybridization controls and incubated overnight with the GeneChip miRNA 3.0 array (Affymetrix) as per manufacturer recommendations. Arrays were scanned using the GeneChip Scanner 3000 7G with autoloader (Affymetrix). Image processing was performed using Affymetrix GeneChip Command Console software followed by analysis with Expression Console software (Affymetrix). Array performance and general data quality were assessed using Signal All, mean background intensity, number of detected probe sets, % P, all probe set mean, all probe set standard deviation, all probe set RLE mean, and % species specific small RNA probe sets detected. All arrays passed standard performance quality thresholds.

### Microarray statistical analysis and data visualization

Individual array data (.cel files) were uploaded to R software package and analyzed using Bioconductor’s Oligo package. Normalization was conducted on all samples in a single set using RMA Background and Quantile Normalization sub-routine. Signal intensities were log2 transformed and probe set values summarized using Median Polish Summarization Method. Final data set included paired samples from exosome cell. Further analysis included 3391 mature and pre-mature human miRNA probe sets. Data visualization tools (e.g., box plot, hierarchical clustering, matrix plots and multi-dimensional scaling) were used to assess the general data quality and outliers. To determine differentially expressed miRNA genes, the mixed model was used to assess differences in miRNA probe expression between exosome samples and cell samples. Unadjusted statistical significance was set at *p* ≤ 0.05 with FDR correction at *p* ≤ 0.10 for multiple testing where relevant. Fold Change values of 2.0 were used as a cut-off to identify up- and down-regulated probes.

Normalized log2 transformed signal data for all human mature miRNAs were imported into Partek Genomics Suite 6.6 software and then filtered to include only miRNA signal data of interest prior to generating individual heat maps. The Tools Discover Hierarchical Clustering subroutine was invoked to create each clustered heat map. Samples and miRNA were clustered using Pearson’s Dissimilarity as the measure of distance with complete linkage.

### Statistical analysis

The results are presented as mean ± standard deviation or SEM (where indicated). The 2-tailed Student *t*-test was used for comparison between groups. A value of *p* < 0.05 was considered statistically significant. To identify linear combinations of serum miRNA markers that could distinguish engrafted from non-engrafted mice, we carried out a logistic regression model of engraftment status at day 14 and day 21 on every possible combination of the markers miR-150, miR-155, and miR-1246. For each of these linear combinations of markers, we examined the corresponding receiver operating characteristic (ROC) curve and estimated the area under the curve (AUC).

### Complete blood count

Blood was drawn from mice retroorbitally into EDTA-treated Microvettes (Sarstedt). Counts were generated using a Hemavet HV950 (Drew Scientific).

## Additional Information

**How to cite this article**: Hornick, N. I. *et al*. Serum Exosome MicroRNA as a Minimally-Invasive Early Biomarker of AML. *Sci. Rep*. **5**, 11295; doi: 10.1038/srep11295 (2015).

## Supplementary Material

Supplementary Information

## References

[b1] Howlader, N. . (eds). SEER Cancer Statistics Review, 1975–2008. http://seer.cancer.gov/csr/1975_2008/ (2011).

[b2] Yin J. A. . Minimal residual disease monitoring by quantitative RT-PCR in core binding factor AML allows risk stratification and predicts relapse: results of the United Kingdom MRC AML-15 trial. Blood 120, 2826–2835, 10.1182/blood-2012-06-435669 (2012). 22875911

[b3] Loken M. R. . Residual disease detected by multidimensional flow cytometry signifies high relapse risk in patients with *de novo* acute myeloid leukemia: a report from Children’s Oncology Group. Blood 120, 1581–1588, 10.1182/blood-2012-02-408336 (2012). 22649108 PMC3429302

[b4] Buonamici S. . Real-time quantitation of minimal residual disease in inv(16)-positive acute myeloid leukemia may indicate risk for clinical relapse and may identify patients in a curable state. Blood 99, 443–449 (2002). 11781223 10.1182/blood.v99.2.443

[b5] Dohner H. . Diagnosis and management of acute myeloid leukemia in adults: recommendations from an international expert panel, on behalf of the European LeukemiaNet. Blood 115, 453–474, 10.1182/blood-2009-07-235358 (2010). 19880497

[b6] Hageman I. M., Peek A. M., de Haas V., Damen-Korbijn C. M. & Kaspers G. J. Value of routine bone marrow examination in pediatric acute myeloid leukemia (AML): a study of the Dutch Childhood Oncology Group (DCOG). Pediatr Blood Cancer 59, 1239–1244, 10.1002/pbc.24124 (2012). 22378688

[b7] Rickmann M. . Monitoring dendritic cell and cytokine biomarkers during remission prior to relapse in patients with FLT3-ITD acute myeloid leukemia. Ann Hematol 92, 1079–1090, 10.1007/s00277-013-1744-y (2013). 23616009 PMC3701796

[b8] Xiao B. . Plasma microRNA signature as a noninvasive biomarker for acute graft-versus-host disease. Blood 122, 3365–3375, 10.1182/blood-2013-06-510586 (2013). 24041574 PMC3821726

[b9] Lim S. H. . Circulating tumour cells and circulating free nucleic acid as prognostic and predictive biomarkers in colorectal cancer. Cancer Lett. 346, 24–33, 10.1016/j.canlet.2013.12.019 (2014). 24368189

[b10] Raghavachari N. . Integrated analysis of miRNA and mRNA during differentiation of human CD34+ cells delineates the regulatory roles of microRNA in hematopoiesis. Exp Hematol 42, 14–27 e11-12, 10.1016/j.exphem.2013.10.003 (2014). 24139908 PMC3878057

[b11] Morris V. A. . MicroRNA-150 expression induces myeloid differentiation of human acute leukemia cells and normal hematopoietic progenitors. PLoS One 8, e75815, 10.1371/journal.pone.0075815 (2013). 24086639 PMC3782459

[b12] Marcucci G. . MicroRNA expression in cytogenetically normal acute myeloid leukemia. N Engl J Med 358, 1919–1928, 10.1056/NEJMoa074256 (2008). 18450603

[b13] Garzon R. . Distinctive microRNA signature of acute myeloid leukemia bearing cytoplasmic mutated nucleophosmin. Proc Natl Acad Sci U S A 105, 3945–3950, 10.1073/pnas.0800135105 (2008). 18308931 PMC2268779

[b14] Wang Y. . MicroRNAs expression signatures are associated with lineage and survival in acute leukemias. Blood Cells Mol Dis 44, 191–197, 10.1016/j.bcmd.2009.12.010 (2010). 20110180 PMC2829339

[b15] Huan J. . RNA trafficking by acute myelogenous leukemia exosomes. Cancer Res. 73, 918–929, 10.1158/0008-5472.CAN-12-2184 (2013). 23149911

[b16] Thery C., Zitvogel L. & Amigorena S. Exosomes: composition, biogenesis and function. Nat. Rev. Immunol. 2, 569–579, 10.1038/nri855 (2002). 12154376

[b17] Simons M. & Raposo G. Exosomes–vesicular carriers for intercellular communication. Curr. Opin. Cell Biol. 21, 575–581, 10.1016/j.ceb.2009.03.007 (2009). 19442504

[b18] Skinner A. M., O’Neill S. L. & Kurre P. Cellular microvesicle pathways can be targeted to transfer genetic information between non-immune cells. PLoS One 4, e6219, 10.1371/journal.pone.0006219 (2009). 19593443 PMC2704871

[b19] Cheng L., Sun X., Scicluna B. J., Coleman B. M. & Hill A. F. Characterization and deep sequencing analysis of exosomal and non-exosomal miRNA in human urine. Kidney Int, 10.1038/ki.2013.502 (2013). 24352158

[b20] Street J. M. . Identification and proteomic profiling of exosomes in human cerebrospinal fluid.J Transl Med 10, 5, 10.1186/1479-5876-10-5 (2012). 22221959 PMC3275480

[b21] Aoki J. . Posttransplantation Bone Marrow Assessment by Quantifying Hematopoietic Cell-Derived mRNAs in Plasma Exosomes/Microvesicles. Clin. Chem., 10.1373/clinchem.2013.213850 (2014). 24452836

[b22] Manterola L. . A small noncoding RNA signature found in exosomes of GBM patient serum as a diagnostic tool. Neuro Oncol, 10.1093/neuonc/not218 (2014). PMC3956347 24435880

[b23] Hong C. S., Muller L., Whiteside T. L. & Boyiadzis M. Plasma exosomes as markers of therapeutic response in patients with acute myeloid leukemia. Front Immunol 5, 160, 10.3389/fimmu.2014.00160 (2014). 24782865 PMC3989594

[b24] Liu J. . Increased Exosomal MicroRNA-21 and MicroRNA-146a Levels in the Cervicovaginal Lavage Specimens of Patients with Cervical Cancer. Int J Mol Sci 15, 758–773, 10.3390/ijms15010758 (2014). 24406730 PMC3907836

[b25] Fayyad-Kazan H. . Circulating miR-150 and miR-342 in plasma are novel potential biomarkers for acute myeloid leukemia. J Transl Med 11, 31, 10.1186/1479-5876-11-31 (2013).

[b26] Sanchez P. V. . A robust xenotransplantation model for acute myeloid leukemia. Leukemia 23, 2109–2117, 10.1038/leu.2009.143 (2009). PMC3659827 19626050

[b27] Agliano A. .Human acute leukemia cells injected in NOD/LtSz-scid/IL-2Rgamma null mice generate a faster and more efficient disease compared to other NOD/scid-related strains. Int. J. Cancer 123, 2222–2227, 10.1002/ijc.23772( 2008). 18688847

[b28] Matsuo Y. . Two acute monocytic leukemia (AML-M5a) cell lines (MOLM-13 and MOLM-14) with interclonal phenotypic heterogeneity showing MLL-AF9 fusion resulting from an occult chromosome insertion, ins(11;9)(q23;p22p23). Leukemia 11, 1469–1477 (1997). 9305600 10.1038/sj.leu.2400768

[b29] Mony U., Jawad M., Seedhouse C., Russell N. & Pallis M. Resistance to FLT3 inhibition in an *in vitro* model of primary AML cells with a stem cell phenotype in a defined microenvironment. Leukemia 22, 1395–1401, 10.1038/leu.2008.125 (2008). 18509353

[b30] Jacamo R. . Reciprocal leukemia-stroma VCAM-1/VLA-4-dependent activation of NF-kappaB mediates chemoresistance.Blood 123, 2691–2702, 10.1182/blood-2013-06-511527 (2014). 24599548 PMC3999754

[b31] Garrido S. M., Appelbaum F. R., Willman C. L. & Banker D. E. Acute myeloid leukemia cells are protected from spontaneous and drug-induced apoptosis by direct contact with a human bone marrow stromal cell line (HS-5). Exp Hematol 29, 448–457 (2001). 11301185 10.1016/s0301-472x(01)00612-9

[b32] Barbash S., Shifman S. & Soreq H. Global coevolution of human MicroRNAs and their target genes. Mol. Biol. Evol. 31, 1237–1247, 10.1093/molbev/msu090 (2014). 24600049

[b33] Rommer A. . Overexpression of primary microRNA 221/222 in acute myeloid leukemia. BMC Cancer 13, 364, 10.1186/1471-2407-13-364 (2013). 23895238 PMC3733744

[b34] Spinello I. .MicroRNA-146a and AMD3100, two ways to control CXCR4 expression in acute myeloid leukemias.Blood Cancer J 1, e26, 10.1038/bcj.2011.24 (2011 ). 22829170 PMC3255264

[b35] Woiterski J. .Engraftment of low numbers of pediatric acute lymphoid and myeloid leukemias into NOD/SCID/IL2Rcgammanull mice reflects individual leukemogenecity and highly correlates with clinical outcome.Int. J. Cancer 133, 1547–1556, 10.1002/ijc.28170 (2013). 23526331

[b36] Reese N. D. & Schiller G. J. High-dose cytarabine (HD araC) in the treatment of leukemias: a review.Curr Hematol Malig Rep 8, 141–148, 10.1007/s11899-013-0156-3 (2013). 23666364

[b37] Kampa-Schittenhelm K. M. . Quizartinib (AC220) is a potent second generation class III tyrosine kinase inhibitor that displays a distinct inhibition profile against mutant-FLT3, -PDGFRA and -KIT isoforms. Mol Cancer 12, 19, 10.1186/1476-4598-12-19 (2013). 23497317 PMC3637582

[b38] Mitchell P. S. . Circulating microRNAs as stable blood-based markers for cancer detection. Proc Natl Acad Sci U S A 105, 10513–10518, 10.1073/pnas.0804549105 (2008). PMC2492472 18663219

[b39] Katsuda T., Kosaka N. & Ochiya T. The roles of extracellular vesicles in cancer biology: towards the development of novel cancer biomarkers.Proteomics, 10.1002/pmic.201300389 (2013). 24339442

[b40] Bazarbachi A. . Human T-cell lymphotropic virus type I-infected cells extravasate through the endothelial barrier by a local angiogenesis-like mechanism.Cancer Res 64, 2039–2046 (2004). 15026341 10.1158/0008-5472.can-03-2390

[b41] Taylor D. D. & Gercel-Taylor C. MicroRNA signatures of tumor-derived exosomes as diagnostic biomarkers of ovarian cancer. Gynecol Oncol 110, 13–21, 10.1016/j.ygyno.2008.04.033 (2008 18589210

[b42] Ferrajoli A. .Prognostic value of miR-155 in individuals with monoclonal B-cell lymphocytosis and patients with B chronic lymphocytic leukemia. Blood 122, 1891–1899, 10.1182/blood-2013-01-478222 (2013). 23821659 PMC3779381

[b43] Kern S. E..Why your new cancer biomarker may never work: recurrent patterns and remarkable diversity in biomarker failures. Cancer Res. 72, 6097–6101, 10.1158/0008-5472.CAN-12-3232 (2012). 23172309 PMC3513583

[b44] Turchinovich A., Samatov T. R., Tonevitsky A. G. & Burwinkel B. Circulating miRNAs: cell-cell communication function? Front Genet 4, 119, 10.3389/fgene.2013.00119 (2013).

[b45] Estey E. H. Acute myeloid leukemia: 2013 update on risk-stratification and management. Am J Hematol 88, 318–327, 10.1002/ajh.23404 (2013). 23526416

[b46] Miraki-Moud F. . Acute myeloid leukemia does not deplete normal hematopoietic stem cells but induces cytopenias by impeding their differentiation. Proc Natl Acad Sci U S A 110, 13576–13581, 10.1073/pnas.1301891110 (2013). 23901108 PMC3746910

[b47] Joosten S. A. . Identification of biomarkers for tuberculosis disease using a novel dual-color RT-MLPA assay. Genes Immun. 13, 71–82, 10.1038/gene.2011.64 (2012). 21956656

[b48] Lim W. S. . Leukemia-selective uptake and cytotoxicity of CPX-351, a synergistic fixed-ratio cytarabine:daunorubicin formulation, in bone marrow xenografts. Leuk Res 34, 1214–1223, 10.1016/j.leukres.2010.01.015 (2010). 20138667

[b49] Rekker K. . Comparison of serum exosome isolation methods for microRNA profiling. Clin. Biochem. 47, 135–138, 10.1016/j.clinbiochem.2013.10.020 (2014). 24183884

