# Supplementary material for: Serum Exosome MicroRNA as a Minimally-Invasive Early Biomarker of AML
Source: Sci Rep. 2015 Jun 12;5:11295. doi: 10.1038/srep11295 (PMC4650871; doi:10.1038/srep11295)
Supplement: Supplementary Information [file srep11295-s1.pdf]

Supplementary Material for

Serum Exosome MicroRNA as a Minimally-Invasive Early Biomarker of AML

Noah I. Hornick, Jianya Huan, Ben Doron, Natalya A. Goloviznina, Jodi Lapidus, Bill H. Chang, Peter Kurre

Fig. S1

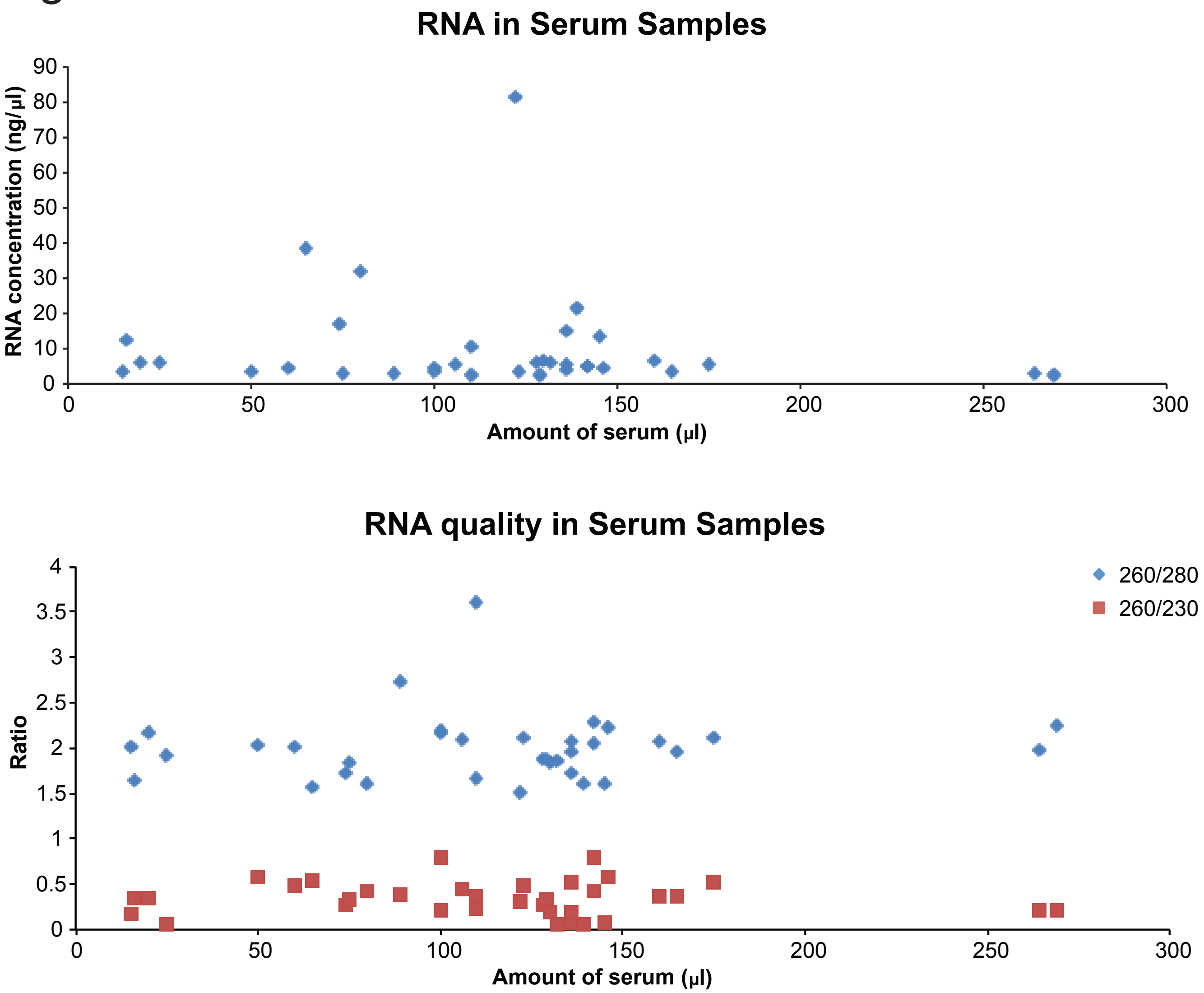

Supplementary Figure S1. Serum exosome RNA yield and quality are independent of serum volume.
Serum was collected from Molm-engrafted or control NSG mice, and exosomes were collected by ExoQuick precipitation. Total RNA was isolated, and concentration, 260/280, and 260/230 values were measured by spectrophotometer.

Fig. S2

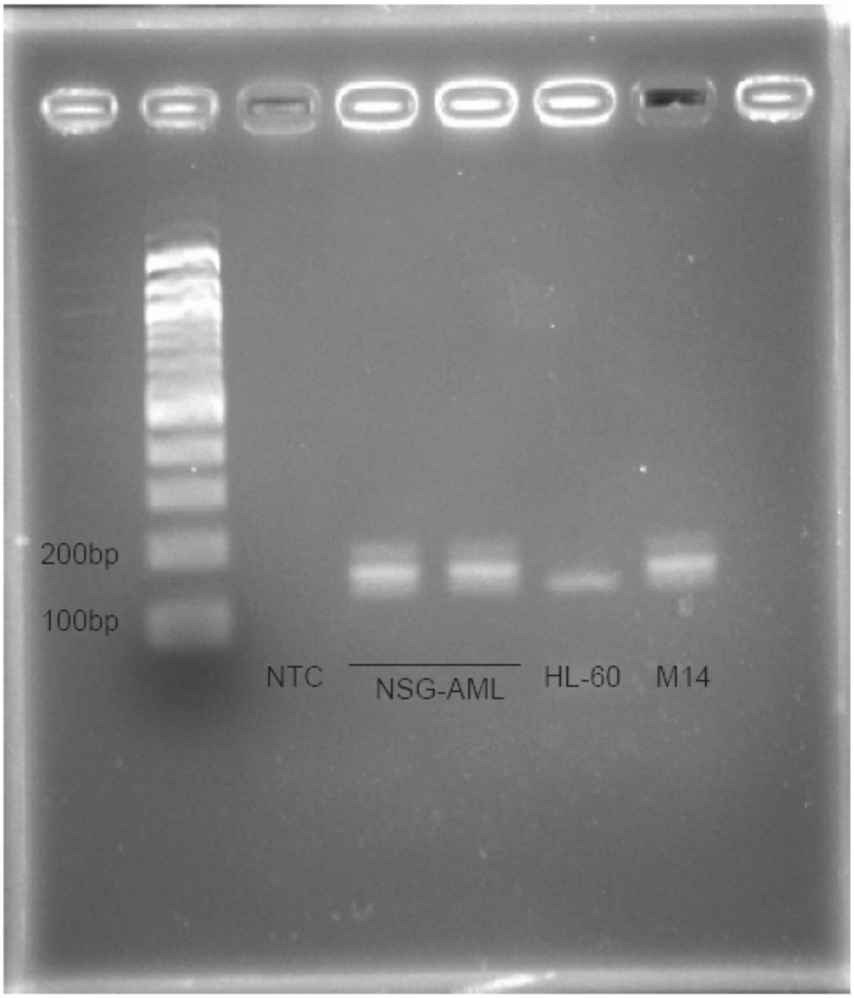

Supplementary Figure S2. PCR for FLT3 in leukemia isolated from NSG xenografts
Molm-14 from engrafted NSG mice retained their characteristic FLT3-ITD mutation, detected by 40 cycles of PCR. NTC: null-template control; NSG-AML: isolated Molm-14; HL-60: cell line exhibiting WT FLT3; M14: Molm-14 stock culture.

Fig. S3

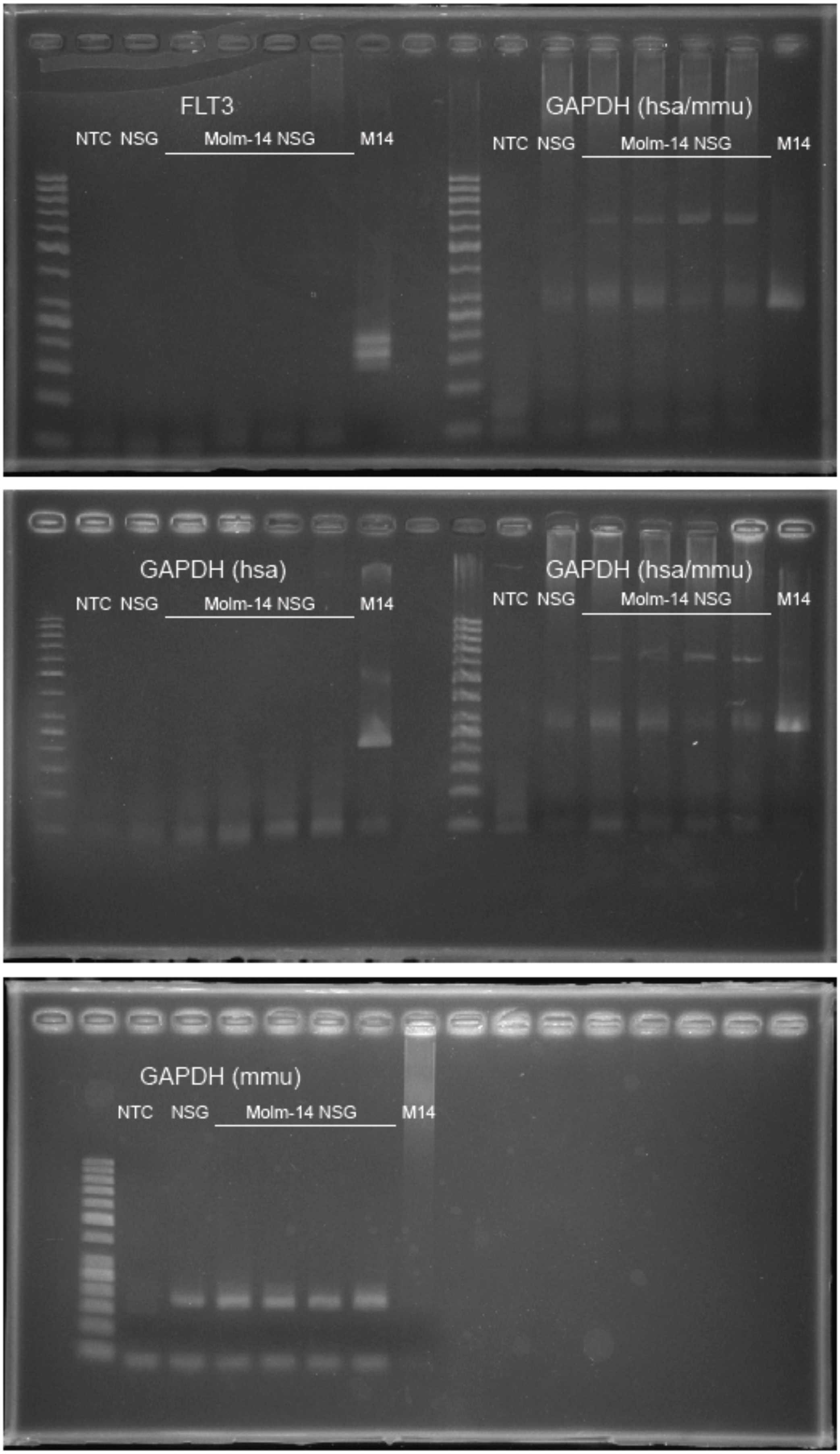
FLT3
NTC NSG Molm-14 NSG M14
GAPDH (hsa/mmu)
NTC NSG Molm-14 NSG M14
GAPDH (hsa)
NTC NSG Molm-14 NSG M14
GAPDH (hsa/mmu)
NTC NSG Molm-14 NSG M14
GAPDH (mmu)
NTC NSG Molm-14 NSG M14

Supplementary Figure S3. PCR for FLT3, GAPDH in serum exosomes isolated from NSG xenografts
Exosomes isolated from the peripheral blood of Molm-14-engrafted NSG mice were tested for human FLT3 and GAPDH and murine GAPDH using 45 cycles of PCR. NTC: null-template control. M14: Molm-14 stock culture. PB: Peripheral blood chimerism; BM: Bone marrow chimerism by flow cytometry for human CD45.

Table S4

	Day 14			Day 21		
**Model**	**n (control)**	**n (AML)**	**AUC**	**n (control)**	**n (AML)**	**AUC**
**1246**	20	18	0.6722	20	18	0.9583
**150**	35	40	0.4600	35	31	0.5217
**155**	34	40	0.6912	34	31	0.8065
**1246, 150**	20	18	0.8083	20	18	0.9667
**1246, 155**	20	18	0.7944	20	18	0.9528
**150, 155**	34	40	0.7463	34	31	0.8643
**1246, 150, 155**	20	18	0.8028	20	18	0.9944

Supplementary Table S4. Comparison of miRNA marker combinations
Logistic regression was used to create weighted linear combinations of serum exosome miRNA markers, which were evaluated for their capacity to discriminate between xenografted mice (*n* in column n (AML)) and unengrafted controls (*n* in column n (control)) at 14 and 21 days post-engraftment. Areas under receiver operating characteristic (ROC) curves are presented as AUC.
